# The Effect of Lifting-and-Thrusting Laser Acupuncture on Electrodermal Activity of Acupoints, Pulse Characteristics, and Brainwave

**DOI:** 10.1155/2023/7342960

**Published:** 2023-04-15

**Authors:** Kun-Chan Lan, Chang-Yin Lee, Kai-Yuan Kuo, Chih-Yu Wang

**Affiliations:** ^1^Department of Computer Science and Information Engineering (CSIE), National Cheng Kung University, Tainan, Taiwan; ^2^The School of Chinese Medicine for Post-Baccalaureate, I-Shou University, Kaohsiung, Taiwan; ^3^Department of Chinese Medicine, E-DA Hospital, Kaohsiung, Taiwan; ^4^Department of Chinese Medicine, E-DA Cancer Hospital, Kaohsiung, Taiwan; ^5^Department of Biomedical Engineering, I-Shou University, Kaohsiung, Taiwan

## Abstract

Acupuncture has been shown as an effective traditional Chinese medicine treatment method, especially for pain relief. Recently, laser acupuncture is becoming increasingly popular, thanks to its noninvasive and painless nature and effectiveness in treating diseases, proven by many studies (for example, some previous studies showed that low-power laser stimulation is able to increase the power of alpha rhythms and theta waves). In our prior work, we developed a novel laser acupuncture model that emulates lifting-and-thrusting operation commonly used in traditional needle acupuncture and showed its benefit in improving cardiac output and peripheral circulation. By extending our previous studies, in this work, we perform extensive experiments to understand the effect of such a system on electrodermal activity (EDA) of acupoints, pulse characteristics, and brainwave, to further verify its efficacy. In particular, we found that laser stimulation could cause significant changes in EDA of acupoints, pulse amplitude, pulse-rate-variability (PRV), and acupoint conductance, as a function of laser power and stimulation time. In addition, laser acupuncture with the lifting-and-thrusting operation has more significant effect on increasing the power of alpha and theta frequency bands as compared to laser acupuncture without the lifting-and-thrusting operation. Finally, given sufficient stimulation time (e.g., > 20 min), the performance of a low-powered laser acupuncture with the lifting-and-thrusting operation could be comparable to that of traditional needle acupuncture.

## 1. Introduction

Needle acupuncture is a common and important treatment method in traditional Chinese medicine (TCM). However, the traditional needle acupuncture treatment might not be suitable for some people due to its intrusive nature which can cause fear of pain or discomfort during the treatment process and introduce some possible potential dangers (such as infection) in the operation. On the other hand, laser acupuncture is an increasingly popular method that aims to stimulates the acupuncture point on the human body with low-intensity, nonthermal laser irradiation. Due to its noninvasive and painless properties, laser acupuncture has become widely accepted by the general public and been considered as an effective and safe treatment method with its efficacy verified by many studies [1–7].

“Lifting and thrusting” is a common technique of traditional needle acupuncture, which plays an important role in acupuncture treatment [8–16]. Researchers have tried to simulate the lifting-and-thrusting technique in laser acupuncture to improve its efficacy [17–20]. We have previously developed a novel device named “Emulated Laser Acupuncture System (ELAS),” which implemented the function of lifting and thrusting in the laser acupuncture system by moving the focused laser spot periodically [21, 22]. In addition, in our prior study, stimulating subject's Neiguan acupoint using laser acupuncture with lifting-and-thrusting function showed a better effect in increasing the skin temperature than that without lifting-and-thrusting function [23, 24]. In this study, we further explored the effects of lifting-and-thrusting function of laser acupuncture on changes in other human physiological parameters such as pulse characteristics, electrodermal activity (EDA) of acupoints, and band power of electroencephalography (EEG), with an aim to provide more evidence for the effectiveness of laser acupuncture with lifting-and-thrusting function.

Prior studies have shown that pulse characteristics and EDA of acupoints can be used to reflect the human physical condition to a certain extent. In particular, pulse diagnosis is one of the diagnostic methods in traditional Chinese medicine and has been practiced for thousands of years. Many studies have shown that the pulse characteristics are able to reflect the states of diseases. For example, Safar et al. observed the increase of aortic pulse wave velocity and brachial pulse pressure can be correlated with the end-stage of renal disease [25]. Gillum et al. showed that an increased resting pulse rate is related to coronary heart disease [26]. Covic et al. reported that a higher aortic pulse wave velocity is associated to a higher risk of coronary artery disease in patients [27]. Finally, Carter compared limbs with arterial occlusive disease (AOD) and limbs without AOD [28] and found that limbs with AOD usually has a lower systolic pressure. On the other hand, electrodermal activity, such as skin resistance, is commonly used for evaluating physiological phenomena [29]. Many prior studies have shown that the skin resistance of acupoints can be used to reflect the patient's health condition. For example, Zhao studied acupoint resistance in patients with epigastric pain [30]. They found that the resistance of patients at Zusanli (ST36) is higher than that of normal people. Szopinski et al. found that the acupoint resistance characteristics of specific locations are dependent on the disease of the corresponding internal organs [31]. Lee et al. compared the acupoint resistance of renal colic patients with healthy controls [32]. The acupoints they chose included the lung (H1), pericardium (H2), heart (H3), spleen (F1), liver (F2), and kidney (F3). They found that the acupoint resistance of the patient group is lower. Prokhorov et al. measured the acupoint resistance from subjects with rheumatoid arthritis [33]. The acupoints they chose are Sanjian (LI3), Erjian (LI2), Guanchong (TH1), Yemen (TH2). They reported that subjects with rheumatoid arthritis have lower acupoint resistance than that of the control group (subjects without rheumatoid arthritis). Lastly, Szopinski and Rayne attempted to detect breast pathology associated with organ electrodermal diagnostics (OED) based on auricular acupuncture points [34]. They confirmed that the change of acupoint resistance can be used to estimate the intensity of a breast pathologic process.

Brainwaves power at different frequency bands may increase or decrease after acupuncture. Li et al. performed laser acupuncture at Neiguan (PC6) and Shenmen (HT7) on normal volunteer subjects and observed changes in the brainwaves after treatment [35]. Kwon et al. measured healthy volunteers' brainwave data and found that the subject's alpha band power at the head position (Fpz, F4, F4, and C3) increased after laser acupuncture treatment [36]. Yu et al. found that the subject's delta band power at Fp1, Fp2, F3, and F4 increased after applying laser acupuncture on Zusanli (ST36) [37]. Kim et al. examined the effects of acupuncture stimulation of PC5 and PC6 and found that alpha band power increased at Fp1, Fp2, C3, C4, T3, T4, O1, and O2 [38]. Li et al. showed that subjects underwent acupuncture at Zusanli (ST36) resulted in increasing delta and theta band power at Fp1, Fp2, C3, C4, T3, T4, O1, and O2 [39].

Several studies have pointed out that stimulating acupoints with acupuncture will change the activity of sympathetic and parasympathetic nerves and achieve the purpose of regulating the autonomic nervous system. Hacker et al. mentioned in their research that acupuncture at Hegu acupoint can simultaneously increase the activity of sympathetic and parasympathetic nerves, and its physiological manifestation is slowing down of heart rate [40]. The research by Li et al. showed that stimulating the Neiguan (PC8) and Hegu (LI4) points in healthy subjects under nonfatigue state would increase the power of LF and HF simultaneously [41]. Manual acupuncture, electromagnetic field, and laser acupuncture stimulating on Xinshu (BL15) acupoint also have impact on the autonomic nerve [42, 43]. Changes in the autonomic nerve activity could affect various physiological parameters such as electrodermal activity, skin temperature, pulse amplitudes, pulse variation, and brainwave.

Although several physiological metrics, such as blood-flow velocity and skin temperature [44–48], have been used to evaluate the efficacy of laser acupuncture, to the best of our knowledge, there are no studies reporting the changes in skin resistance and pulse characteristics after laser acupuncture. In this work, we investigate whether laser acupuncture, with or without lifting-and-thrusting function, can achieve a similar effect as the needle acupuncture by observing the changes in acupoint conductance, pulse-wave characteristics, heart rate variability (PRV), and band power of electroencephalography (EEG).

## 2. Materials and Methods

### 2.1. System Architecture and Sensors

**Figure 1:**
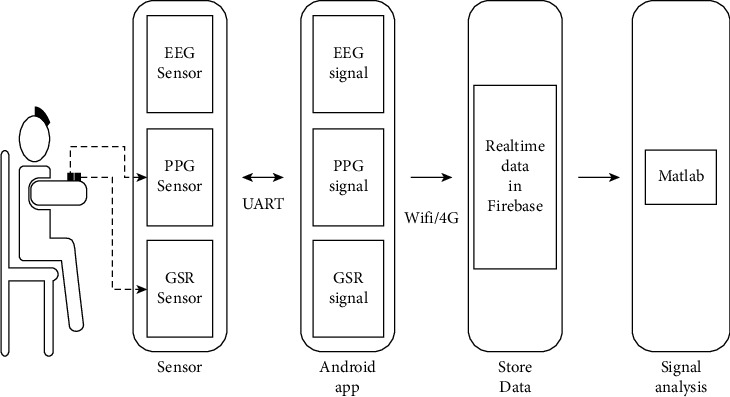
System architecture.

Figure 1 shows the diagram of our system architecture. The PPG sensor measured the “guan” pulse signals on the left and right wrist. The GSR sensor was used to measure the skin resistance at acupoints Hegu (LI4) and Neiguan (PC6). The acquired signal was collected by a smart phone and uploaded to Firebase through an app on the Internet. Finally, we download the data to a personal computer (PC) for the calculations and analyses with MATLAB R2018b.

**Figure 2:**
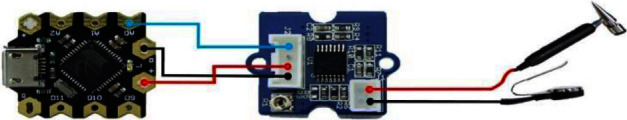
Circuit diagram of the GSR recorder.

**Figure 3:**
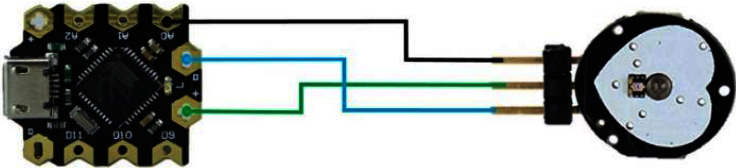
Circuit diagram of the PPG recorder.

**Figure 4:**
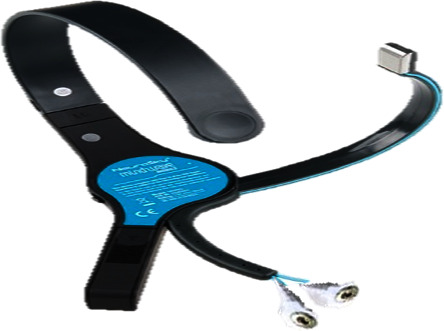
NeuroSky Mindwave Mobile+ with ear clip replaced with electrode.

Figure 2 provides a circuit diagram of GSR (galvanic skin response) recorder. The method described in [49] was used to measure the skin response, and the sample rate was 200 Hz.  Figure 3 is a circuit diagram of the PPG recorder, which records data at a sampling rate of 25 Hz. The method described in [49] was used to measure the PPG data. The PPG recorder comprised the Arduino Bluno Beetle and the PPG sensor, which absorbed light energy on the skin area through a light sensing element.  Figure 4 shows a NeuroSky MindWave Mobile+ (sampling rate: 512 Hz, resolution: 12 bits, and 3–100 Hz bandpass filter and remove 60 Hz), which was used to measure the EEG signals.

### 2.2. Signal Analysis

The original PPG signal was filtered, and the low-frequency DC drift noise (below 0.15 Hz) and high-frequency noise (above 20 Hz) were removed. A self-developed program was used for finding the systolic peak, following the method in [49]. This method consists of three stages: signal preprocessing (including bandpass filtering and squaring), generation of blocks of interest using two moving averages, and classification-based adaptive thresholding [50].

We analyzed five frequency bands in the collected EEG signals, including delta (1–4 Hz), theta (4–8 Hz), alpha (8–12 Hz), beta (12–30 Hz), and gamma (30–50 Hz). The brainwave data were converted into frequency domain with a fast Fourier transform (FFT); then, the values in each frequency range were averaged to obtain the power in delta, theta, alpha, beta, and gamma bands, respectively.

### 2.3. Experiment

**Figure 5:**
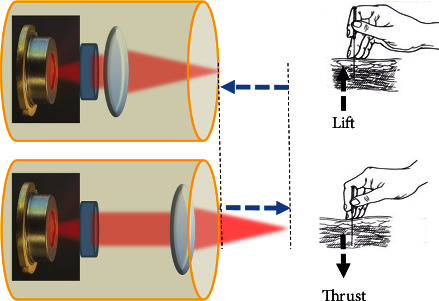
Principle of lift-and-thrust function in laser acupuncture [23].

In this study, GSR and PPG were used to collect physiological signals. In addition, two types of laser acupunctures were used for efficacy verification: an emulated laser acupuncture system (ELAS) and a cordless laser wrap system (emLas, Konftec Corporation, Taiwan). The ELAS is a self-developed laser acupuncture system with programmable automatic lift-and-thrust function [23]. It uses a laser diode with wavelength of 808 nm and focused light spot moving in a range of 2 cm with a frequency of 1 Hz.  Figure 5 illustrates how the ELAS laser acupuncture system implement the function of lifting and thrusting.

The duration and power of the emLas system can be set up with buttons on the laser pen. The wavelength of this laser pen is 660 nm [51].

**Figure 6:**
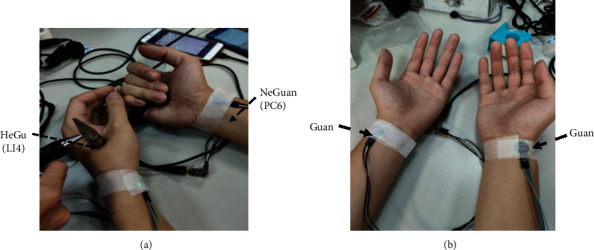
Methods for GSR and PPG measurement. (a) GSR measurement. (b) PPG measurement.

Figure 6(a) shows the experiment for acupoint impedance measurement. The measurement device consisted of a clip (as the reference) and a probe. The subject holds the clip in the palm of his hand, and position the probe on the acupoint to measure the impedance. The acupoints used in our experiments include Hegu (LI4) and Neiguan (PC6).  Figure 6(b) shows the experiment for the PPG measurement. The PPG sensor is placed on the “Guan” position, which is often used by a Chinese medicine doctor to observe a patient's pulse.

The experiment was conducted in a quiet room with a temperature between 23°C and 26°C, so that the subjects felt comfortable and relaxed during the experiment. During the experiment, the subjects were instructed to sit upright with their arms flat on the table, avoid talking or moving their arms.

**Figure 7:**
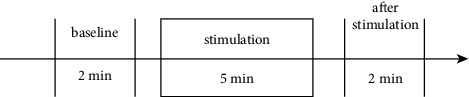
Experimental procedure.

Figure 7 illustrates the experiment procedure. First, the body state before laser stimulation was first measured (two minutes in total, as baseline). Measurements included the subject's acupoint impedance at Hegu and Neiguan, PPG signal measurements at the “Guan” position on the left and right wrists, and EEG measurements using NeuroSky MindWave+. During the period of laser acupuncture stimulation, PPG data and EEG data were continuously recorded, while acupoint impedances are only measured before and after the laser stimulation, due to that laser acupuncture stimulation and acupoint impedance measurement cannot be performed at the same time. Finally, after five minutes' laser acupuncture stimulation, the acupoint impedance, PPG, and EEG data were measured for another two minutes.

A total of 30 healthy subjects, aged 20–24 years, were recruited for this study. This experiment was approved by the Institutional Review Board (IRB) of EDA Hospital, Kaohsiung, Taiwan (IRB number. EMRP-105-005(RIV)). Before experiment, an explanation was given, and a consent form was signed by the subject. The inclusion and exclusion criteria for the subjects are described as follows:

(i) Inclusion criteria: the subjects were healthy adults over 20 years old. Both male and female are available.
(ii) Exclusion criteria are as follows:
  - People with chronic diseases, such as heart disease, hypertension, diabetes, epilepsy, and cancer, are not suitable for this trial
  - Unhealthy conditions caused by nondisease factors, such as drug poisoning, poor nutritional status, genetic medical history, and allergies, are not suitable for this trial
  - People who are not suitable for acupuncture, such as those with wounds near the needle site, fasting, fatigue, severe sweating, and profuse sweating after exercise, are not suitable for this trial
  - People who are unwilling to sign the subject's consent form, or who cannot joint the test completely, are not suitable to participate in this trial

## 3. Results

In this study, we compared the efficacy of the following acupuncture modes: (a) laser acupuncture without lifting-and-thrusting function; (b) laser acupuncture with lifting-and-thrusting function; (c) traditional needle acupuncture without lifting and thrusting. The acupoints tested include Hegu and Neiguan. We investigate the following three parameters for the performance evaluation of laser acupuncture. The result of needle acupuncture serves as a baseline for comparison. The needle acupuncture was performed by a TCM practitioner, and the needle was retained for 20 minutes.

- Effect of laser power: A laser acupuncture of 660 nm wavelength with two kinds of power (250 mW and 300 mW, respectively) were used (no lifting-and-thrusting function). The duration of laser stimulation is set to 5 minutes.
- Effect of stimulation duration: An ELAS laser acupuncture system (with the function of lifting and thrusting) is used. The laser wavelength is 808 nm. The rate of lifting and thrusting is 1 Hz, and the laser power is set to 120 mW. Two kinds of stimulation duration were employed (15 minutes and 20 minutes, respectively).
- Effect of lifting-and-thrusting: An ELAS laser acupuncture system as described was used. The lifting-and-thrusting function could be turned on or off on this system. The stimulation duration could be adjusted from 5 minutes to 20 minutes.

### 3.1. The Effects on Electrodermal Activity (EDA) of Acupoints

**Figure 8:**
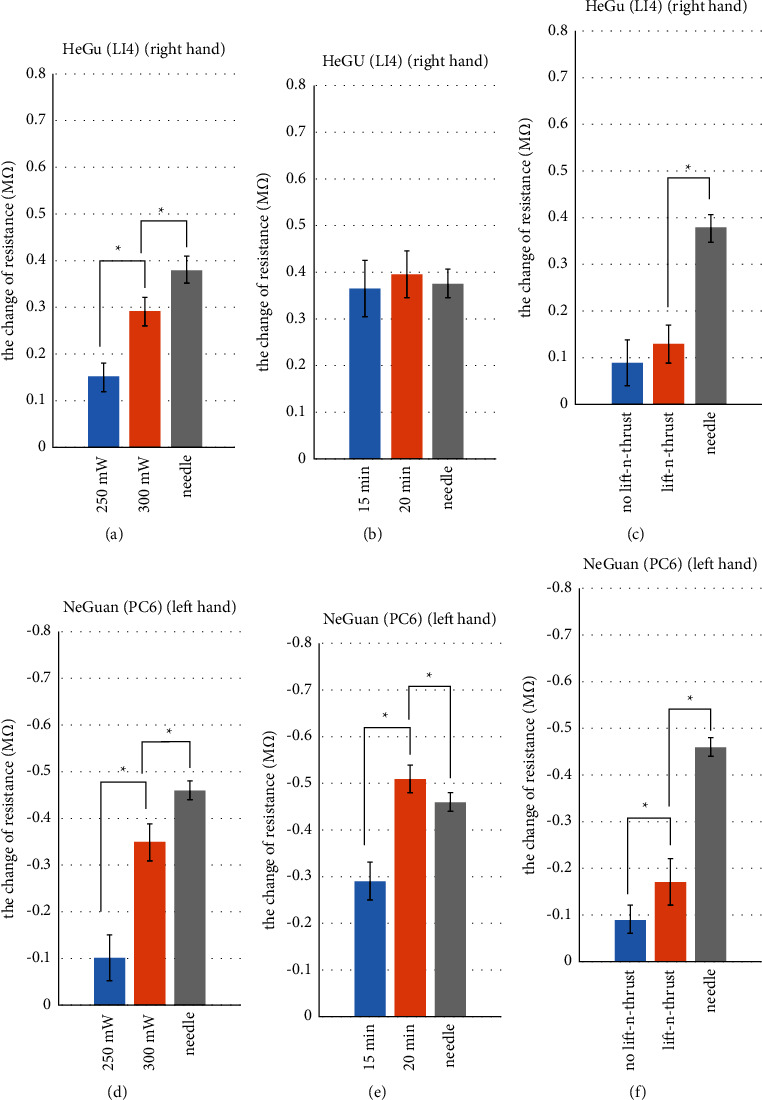
Change of EDA underwent laser acupuncture or needle acupuncture stimulation on Hegu (LI4) and Neiguan (PC6). Asterisks (^*∗*^) indicate significant differences by two-sided *t*-test (*p* < 0.05).

In this section, we focused on whether laser acupuncture can achieve similar effects to needle acupuncture on electrodermal activity (EDA) of acupoints. Before each laser acupuncture or needle acupuncture stimulation, skin resistance at acupoints was acquired as baseline. The measurement results in the experiment were subtracted from the baseline, to calculate the changes after the acupuncture operation.  Figure 8 shows the change of EDA (i.e., skin resistance at the acupoint) underwent laser acupuncture or needle acupuncture stimulation on Hegu (LI4) and Neiguan (PC6). Looking at the results, we observed the following points:

- In the stimulation of Hegu point with laser acupuncture and needle acupuncture, the EDA showed an upward trend (i.e., the skin resistance increases after the acupoint stimulation); while in the stimulation of Neiguan point, the EDA showed a downward trend (i.e., the skin resistance decreases after the acupoint stimulation). Such a phenomenon, as far as we know, was first observed and is probably related to the characteristics of the associated meridians (e.g., Yang meridian vs. Yin meridian).
- Higher laser power results in the greater change in EDA (Figures 8(a) and 8(d)). This observation is consistent with previous studies [45].
- With sufficient stimulation time (e.g., 20 minutes), laser acupuncture with lifting-and-thrusting function can possibly lead to a greater change in EDA than the traditional needle acupuncture (Figures 8(b) and 8(e)).
- The changes of EDA caused by laser acupuncture with lifting-and-thrusting function were significantly higher than those without the lifting-and-thrusting function, based on Figures 8(c) and 8(f).

### 3.2. The Effects on Pulse Characteristics

In this section, we examined whether laser acupuncture can achieve similar effects to needle acupuncture on pulse characteristics. Photoplethysmography (PPG) signals were first acquired in this experiment. PPG data are then used to compute time domain features such as pulse amplitude and pulse rate variability (PRV), as well as the frequency domain features, such as the low-frequency power (namely LF, frequency activity in the 0.04–0.15 Hz range) and high-frequency power (HF, frequency activity in the 0.15–0.40 Hz range).

Before the acupuncture stimulation, time- and frequency-domain features of PPG data were calculated to serve as the baseline. The measurement results in the experiment were subtracted from the baseline, to calculate the changes after the acupuncture operation.

#### 3.2.1. The Effects on Time Domain Features of Pulse Wave

**Figure 9:**
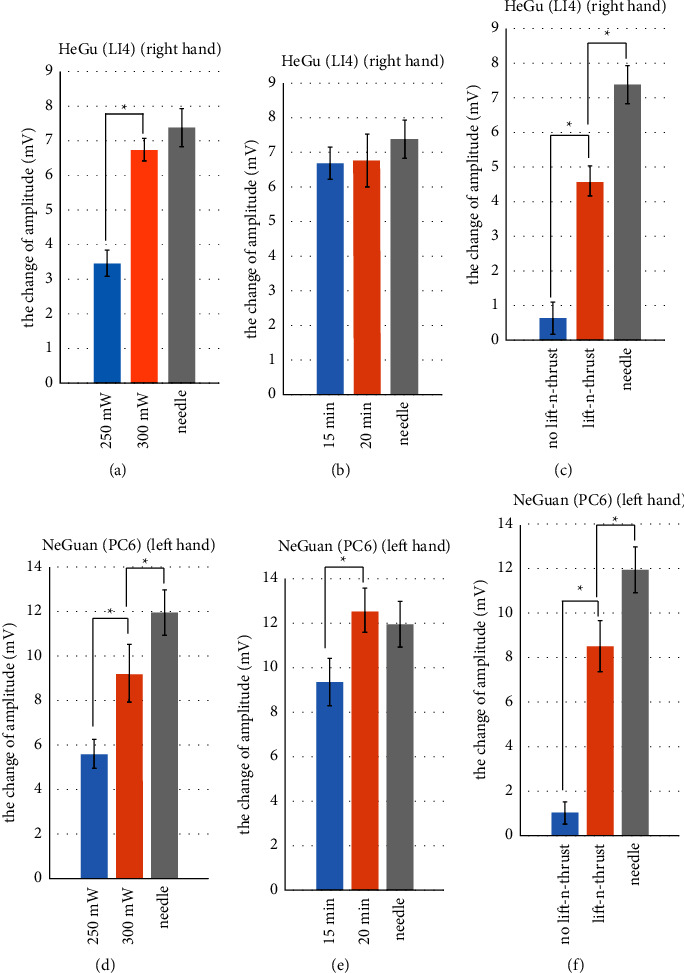
Change of pulse amplitudes underwent laser acupuncture or needle acupuncture stimulation on Hegu (LI4) and Neiguan (PC6). Asterisks (^*∗*^) indicate significant differences by two-sided *t*-test (*p* < 0.05).

**Figure 10:**
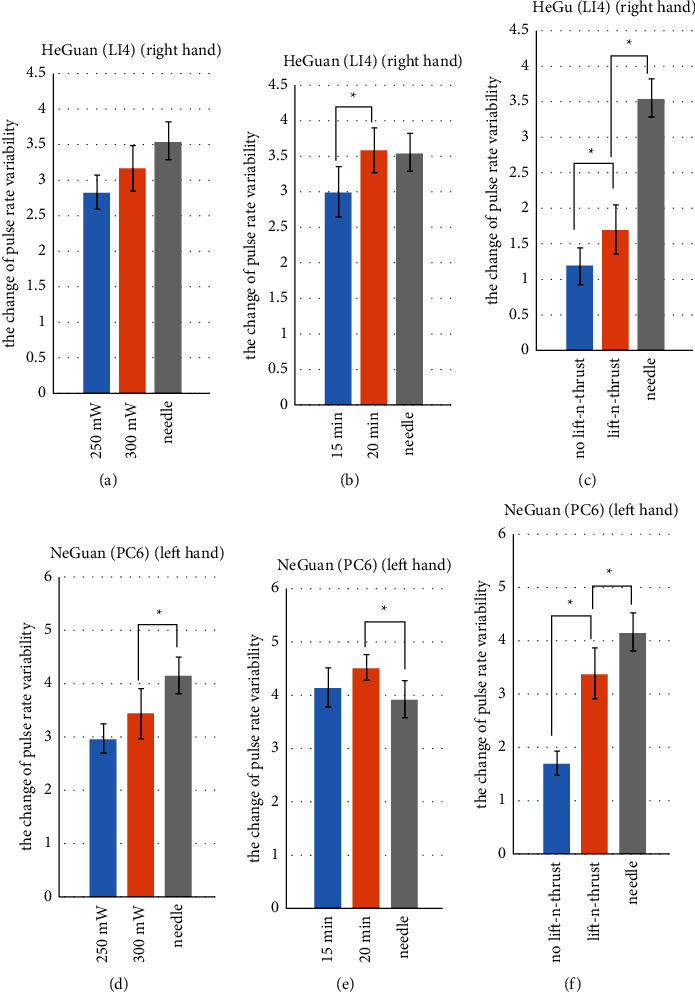
Change of pulse rate variability (PVR) underwent laser acupuncture or needle acupuncture stimulation on Hegu (LI4) and on Neiguan (PC6). Asterisks (^*∗*^) indicate significant differences by two-sided *t*-test (*p* < 0.05).

Figures 9 and 10 show change of pulse amplitude and pulse rate variability (PVR) underwent laser acupuncture or needle acupuncture stimulation on Hegu (LI4) and Neiguan (PC6). These results suggestions are described as follows:

- Both the pulse amplitudes and PVR showed an upward trend (i.e., they increases after the acupuncture stimulation) for Hegu and Neiguan. Note that, the change when stimulating Neiguan point, generally speaking, is higher than that when stimulating the Hegu point, with either the needle acupuncture or laser acupuncture. This phenomenon might be related to characteristics of different meridians these two acupoints associated to. Some prior studies [52] have shown that stimulating Neiguan (PC6) acupoint might have the effect of dilating blood vessels, increasing stroke volume and promoting peripheral blood circulation.
- Similar results are found and are consistent with the results described in Section 3.1. Specifically, higher laser power results in the greater change in pulse amplitude and PRV (Figures 9(a) and 9(d); Figures 10(a) and 10(d)). With sufficient stimulation time (e.g., 20 minutes), laser acupuncture with lifting-and-thrusting function can possibly lead to a greater change in pulse amplitude and PRV than the traditional needle acupuncture (Figures 9(b) and 9(e), Figures 10(b) and 10(e)). And finally, the changes of pulse amplitude and PRV caused by laser acupuncture with lifting-and-thrusting function were significantly higher than those without the lifting-and-thrusting function (Figures 9(c) and 9(f); Figures 10(c) and 10(f)).

#### 3.2.2. The Effects on Frequency Domain Features of Pulse Wave

**Figure 11:**
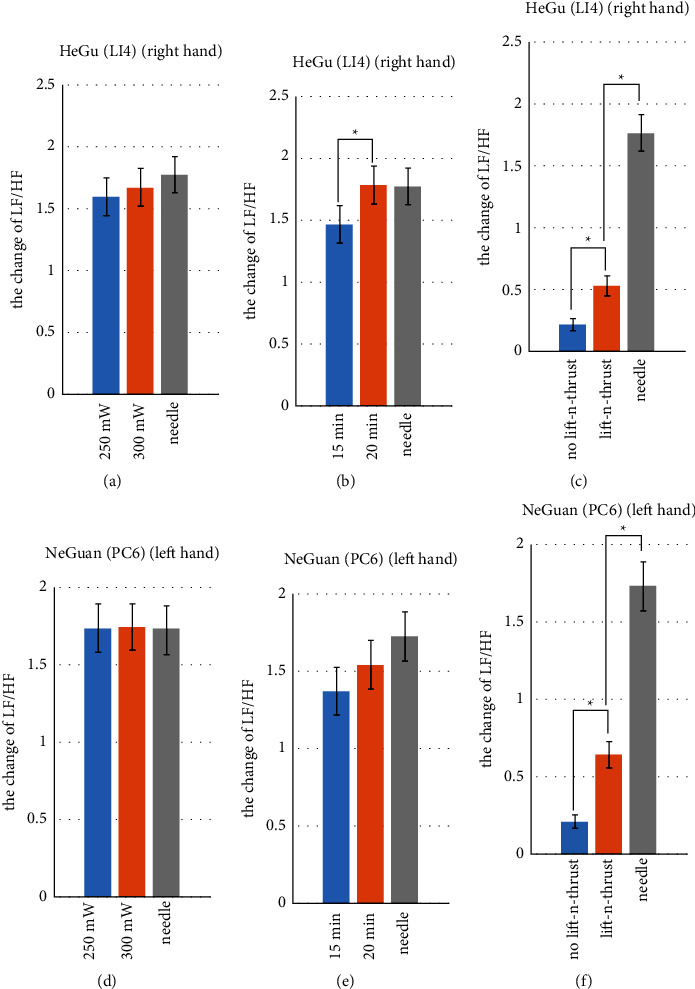
Change of the ratio between the low-frequency power and high-frequency power (LF/HF) underwent laser acupuncture or needle acupuncture stimulation on Hegu (LI4) and Neiguan (PC6). Asterisks (^*∗*^) indicate significant differences by two-sided *t*-test (*p* < 0.05).

Figure 11 shows change of the ratio between the low-frequency power and high-frequency power (LF/HF) underwent laser acupuncture or needle acupuncture stimulation on Hegu (LI4) and Neiguan (PC6). In general, these results are consistent with the results from the analysis of time domain features of pulse waves, as described above. In summary, the LF/HF ratio increases after the acupuncture stimulation for both Hegu and Neiguan. In addition, higher laser power results in the greater change in LF/HF (Figures 11(a) and 11(d)). With sufficient stimulation time (e.g., 20 minutes), the effect of laser acupuncture with lifting and thrusting function is comparable to that of the traditional needle acupuncture (Figures 11(b) and 11(e)). Finally, the changes of LF/HF caused by laser acupuncture with lifting-and-thrusting function were significantly higher than those without lifting-and-thrusting function (Figures 11(c) and 11(f)).

### 3.3. Acupuncture Effects on Brainwave

**Figure 12:**
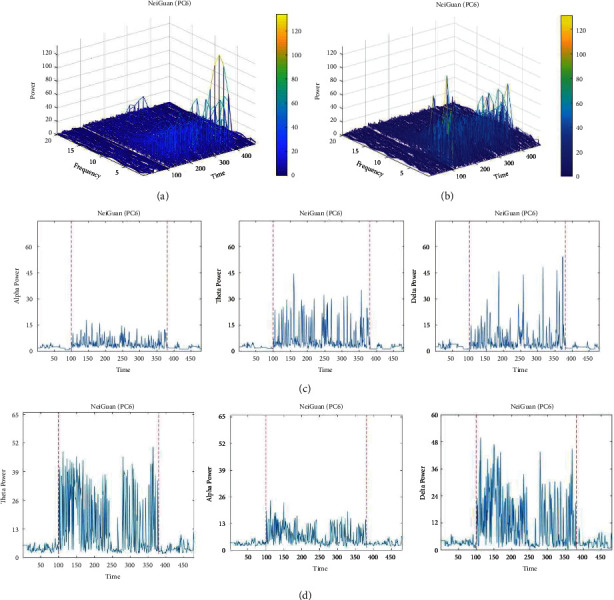
EEG power for various frequencies recorded from one subject (subject 7). (a) Stimulation on Neiguan without lifting and thrusting; (b) stimulation on Neiguan with lifting and thrusting; (c) band power of delta, theta, and alpha bands when stimulation on Neiguan without lifting and thrusting; (d) band power of delta, theta, and alpha bands when stimulation on Neiguan with lifting and thrusting.

In this section, we studied the effect of laser acupuncture on brainwaves on stimulating Hegu and Neiguan points. The EEG signals were recorded with a commercial device (MindWave Mobile+, NeuroSky Inc. San Jose, USA). The laser acupuncture, with or without lifting-and-thrusting function, was applied on Hegu and Neiguan acupoint for 6 minutes, respectively. The EEG measurements were collected before and after the acupuncture stimulation, and the band power for each frequency was computed. The stimulation starts at the 100^th^ second and ends at the 380^th^ second.  Figure 12 illustrates the band power of various frequencies in EEG signal at different times, when stimulating Neiguan with or without lifting-and-thrusting function. We observed increased power in lower frequency bands (delta, theta, and alpha) when the acupoint was stimulated with or without lifting-and-thrusting function. In addition, the increases of band power caused by laser acupuncture with lifting-and-thrusting function were significantly higher than those without lifting and thrusting function (Figures 12(c) and 12(d)). The results from stimulating Hegu are similar to the results for Neiguan (as described above), so that we do not show them here for brevity.

## 4. Discussion

Due to that needle acupuncture with lifting and thrusting could result in pain to patients and more labor work to acupuncturists, in practice, needle acupuncture is usually not performed with long lifting-and-thrusting operation. In this study, for needle acupuncture experiments, no lift-thrust operation was performed together with the needle acupuncture, and the needle was retained for 20 minutes. On the other hand, given that the advantage of low-power laser acupuncture is that it does not introduce pain to the patient; we can simulate the lift-thrust operation continuously for 5 minutes or even longer time. Our results suggest that the performance of laser acupuncture with lifting-thrusting function enabled for 20 minutes is close to that of needle acupuncture (without lift-thrust operation).

**Figure 13:**
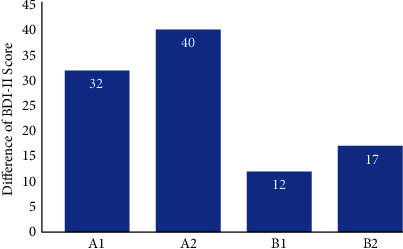
The difference of BDI-II scores (scores after laser acupuncture stimulation minus score before laser acupuncture stimulation) with lifting-and-thrusting function (subjects A1 and A2) and without lifting-and-thrusting function (subjects B1 and B2).

Laser acupuncture could affect the sympathetic and parasympathetic nerves of the human body and regulate the autonomic nerves, therefore affect the physiological parameters such as electrodermal activity of acupoints, pulse amplitudes, pulse variation, and brainwave. In this study, we focus on the effect of laser acupuncture with lifting and thrusting on the changes of these physiological parameters. The subjects of the study are all healthy adults, and the effect of laser acupuncture with lifting and thrusting on disease treatment (e.g., pain relief) was not studied. On the other hand, we are currently conducting a clinical trial on the treatment of depression using laser acupuncture with lifting and thrusting to stimulate Shenmen (HT7) and Taichong (LR3) of the patients, respectively. The severity of depression patients was evaluated using the Beck Depression Inventory (BDI-II). The subjects received laser acupuncture stimulation for 5 minutes each time, twice a week for 8 weeks, with a total of 16 courses of treatment.  Figure 13 shows some initial results about the difference of BDI-II Scores (scores after laser acupuncture stimulation minus score before laser acupuncture stimulation). Greater difference indicates better improvement of depression. The score difference of those patients treated with lifting-and-thrusting function is generally higher than that of those treated without lifting-thrusting function. According to the literature, heart rate variability (pulse rate variability) can be used as an indicator of the severity of depression. In the study of Van der Kooy et al., it was mentioned that the elderly with major depression had significantly lower heart rate variability than those without depression [53]. The research of Hartmann et al. also shows that there are significant differences between the depressed patients and healthy subjects in frequency domain parameters such as HF power and LF power and in time domain parameters such as SD1 and RMSSD [54]. However, our research on laser acupuncture with lifting-and-thrusting function in the treatment of depression focuses on the results of the Beck Depression Inventory (BDI-II); thus, we did not include other metrics in this study. Since combining these physiological parameters with disease treatment is a topic worthy of investigation, we will consider including them in our future studies.

**Figure 14:**
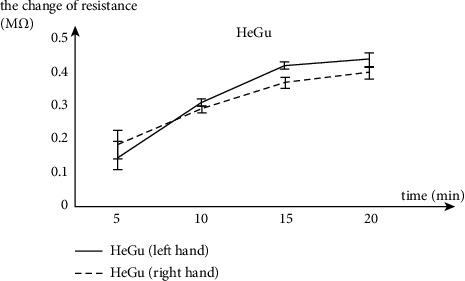
The effect of changing the stimulation duration of laser acupuncture on skin resistance of acupoint (Hegu).

In this study, we observe that a higher laser power generally will introduce a greater change in EDA and pulse characteristics, as demonstrated in Figures 8–11. In addition, we find that a longer stimulation time will generate similar effects (i.e., a longer stimulation time will introduce a greater change in EDA and pulse characteristics) with or without the lifting and thrusting operation. As an example, here, we show the effect of stimulation duration on the change of skin resistance of Hegu, as shown in Figure 14. These observations are consistent with the prior study [45] which showed that the power and stimulation duration are important parameters of laser acupuncture.

A total of 30 healthy subjects were recruited in this study. In terms of acupoints, two acupoints, Hegu and Neiguan, were selected for our experiments. The parameters of laser acupuncture include stimulation duration, stimulation power, and the operation methods (i.e., with or without lifting and thrusting). In the measurement of human physiological parameters, we studied the changes of skin resistance at acupoints, pulse amplitude, pulse rate variability (PRV), and LF/HF. Within the abovementioned scope, our experiments demonstrate that laser acupuncture with a lifting-and-thrusting function is comparable to the traditional needle acupuncture, given sufficient stimulation time or power. More works are needed to further verify the efficacy of laser acupuncture with a lifting-and-thrusting function though. For example, our subject size is small and we did not include a placebo group in our experiments. In terms of laser acupuncture parameters, the influence of laser rate density (output power/cm^2^), frequency, and wavelength need to be examined. In terms of physiological parameters, the systolic blood pressure, diastolic blood pressure, and pulse wave velocity of the pulse can be measured. In terms of acupoint selection, measurements of other acupoints can be added. Finally, most of our recruited subjects are healthy young adults. Improving the heterogeneity of subjects (e.g., including subjects with specific diseases or different age groups) in our future work might provide some new insight.

## 5. Conclusion

This study explored the effects of laser acupuncture with the lifting-and-thrusting function (ELAS) on various physiological parameters such as skin resistance, pulse amplitude, pulse rate variability (PRV), LF/HF, and EEG frequency band power. We showed that, with sufficient stimulation power or stimulation duration, the performance of ELAS could be comparable with the traditional needle acupuncture. Overall, the changes of these physiological parameters caused by laser acupuncture with lifting-and-thrusting function were significantly higher than those without the lifting-and-thrusting function. In addition, a higher laser power or a longer stimulation duration generally will introduce a greater change in these physiological parameters. Finally, we observed increased power in lower EEG frequency bands (delta, theta, and alpha) when the acupoint was stimulated by the laser acupuncture with or without lifting-and-thrusting function.

## Data Availability

The data used to support the findings of this research are available on request to the corresponding author.
